# Using Computerised Gait Analysis to Assess Changes After Rehabilitation in Knee Osteoarthritis: A Systematic Review and Meta-Analysis of Gait Speed Improvement

**DOI:** 10.3390/medicina61091540

**Published:** 2025-08-27

**Authors:** Mihaela Minea, Sermina Ismail, Lucian Cristian Petcu, Andreea-Dalila Nedelcu, Adina Petcu, Alexandra-Elena Minea, Mădălina-Gabriela Iliescu

**Affiliations:** 1 Faculty of Medicine, Doctoral School, Ovidius University of Constanta, 1 University Alley, Campus-Corp B, 900470 Constanta, Romania; mihaela.minea@365.univ-ovidius.ro (M.M.); lucian-cristian.petcu@365.univ-ovidius.ro (L.C.P.); dalila.nedelcu@365.univ-ovidius.ro (A.-D.N.); 2 Balneal and Rehabilitation Sanatorium of Techirghiol, Victor Climescu Street 34–40, 906100 Techirghiol, Romaniaș; 3 Faculty of Dental Medicine, Ovidius University of Constanta, 7 Ilarie Voronca Street, 900178 Constanta, Romania; 4 Faculty of Pharmacy, Ovidius University of Constanta, 1 University Alley, Campus-Corp B, 900470 Constanta, Romania; adina.petcu@365.univ-ovidius.ro; 5 Faculty of Medicine, Carol Davila University of Medicine and Pharmacy, Dionisie Lupu Street, no. 37, Sector 2, 050474 Bucharest, Romania; alexandra-elena.minea0720@stud.umfcd.ro

**Keywords:** knee osteoarthritis, rehabilitation, computerised gait analysis, systematic review, meta-analysis, gait speed

## Abstract

*Background and objectives*: Knee osteoarthritis (KOA) is a degenerative joint disorder often associated with altered gait mechanics. This systematic review aims to evaluate the effect of rehabilitation treatment on walking, with a focus on gait speed. *Material and methods*: A systematic search was conducted in PubMed, Scopus, Web of Science, Cochrane, and PEDro databases, following the PRISMA (Preferred Reporting Items for Systematic Reviews and Meta-Analyses) methodology. Randomised controlled trials published in English between 2015 and 2025, involving patients with KOA undergoing rehabilitation and using computerised gait measurements, including 3D motion capture, force plates, and instrumented treadmills, were included. A meta-analysis was conducted on the selected studies assessing gait speed, with the risk of bias being evaluated using the Cochrane Risk of Bias 2 tool for RCTs. *Results:* Out of 2143 articles, 7 studies met the inclusion criteria. These studies showed increased gait speed in patients with KOA following exercise therapy, various walking training programmes, massage, or dietary interventions. The meta-analysis revealed a standardised mean difference (SMD) of 1.807, with a 95% confidence interval (CI) of [1.637, 1.977] (*p* < 0.001). The interventions were associated with improvements in walking parameters and quality of life, as well as reductions in pain and fall risk. Substantial heterogeneity was noted, likely due to variations in intervention types and study populations. *Conclusions:* The results suggest an overall improvement in gait speed in the intervention groups compared to the control groups. However, only a limited number of studies have investigated the effects of physiotherapy, bath therapy, or mud therapy on gait mechanics in patients with KOA.

## 1. Introduction

### 1.1. The State of Knowledge

Knee osteoarthritis (KOA) is characterised by pain, stiffness, dysfunction, and changes in gait, leading to disabilities [1] and impairing the patient’s quality of life [2]. Patients with knee osteoarthritis (KOA) typically exhibit altered gait characteristics compared with healthy individuals, characterised by changes in temporospatial, kinetic, and kinematic parameters [1]. There is a reduced self-selected walking speed, decreased stride length and height, shorter swing phase, and shorter duration of single-leg support. KOA patients tend to have an increased gait cycle duration, spend a greater proportion of time in the stance phase, and exhibit prolonged double-leg support time [3,4], reflecting compensatory strategies aimed at reducing pain and joint loading during ambulation [5]. They also show lower peak knee flexion angles and a reduced knee flexion range of motion compared with healthy controls, while their peak knee adduction angles are higher. Moreover, patients with KOA have significantly decreased peak knee flexion moments, but increased peak knee adduction moments (KAMs), peak knee rotation moments (KRMs), and greater KAM and KRM impulses [3].

The Osteoarthritis Research Society International (OARSI) recommends five performance-based tests to assess physical function in individuals with knee osteoarthritis: the 30 s chair stand test, the 40-metre fast-paced walk test, the stair climb test, the Timed Up-and-Go test, and the 6 min walk test. Among these, the first three tests—the chair stand test, the fast-paced walk test, and the stair climb test—are recommended as the minimum core set of performance-based outcome measures to use in osteoarthritis research and clinical practice [6].

In KOA studies, standard scales assess pain intensity, physical function, overall patient health perception, and quality of life [7]. Tools like Western Ontario and McMaster University’s Osteoarthritis Index (WOMAC) [8] and the Knee Injury and Osteoarthritis Outcome Score (KOOS) [9] evaluate function, while pain is measured by scales like the Likert scale, Numerical Rating Scale (NRS), and Visual Analogue Scale (VAS). Patient global assessment captures the overall impacts and quality of life and is measured by instruments such as the Short Form-36 (SF-36) [10], EQ-5D [11], health assessment questionnaire disability index (HAQ-DI) [12], and Health Utilities Index (HUI) [13]. Another scale that is used to assess lower limb function in KOA is the Short Physical Performance Battery (SPPB), which includes balance tests, the chair stand test, and a 4-metre walking speed test and yields a total score ranging from 0 to 12, with higher scores indicating better physical performance [14].

Computerised gait analysis is a quantitative assessment method that includes technologies like 3D motion systems, force platforms, inertial sensors, and digital setups for objective measurement of temporospatial, kinetic, and kinematic walking parameters [15]. Various systems are currently employed for this type of gait assessment. The gold standard in research is systems that utilise 3D optical capture with infrared cameras and reflective markers that are placed on the body, producing highly accurate 3D kinematic data (angles, trajectories, and velocities). However, they are costly and require a specialised laboratory. Force platforms measure ground reaction forces during movement, with sensors embedded in the floor or walking strips [15]. They are used to calculate kinetic parameters such as knee adduction moment (KAM), ground reaction forces (GRFs), and knee adduction angular impulse (KAAI), as well as some temporospatial parameters, including gait speed [16]. Instrumented treadmills are equipped with integrated pressure sensors or force platforms. They allow for long-term gait analysis under standardised conditions and can also include real-time visual feedback. Inertial sensor-based systems are equipped with wearable sensors (accelerometers, gyroscopes) that are placed on the body and limbs [17]. They measure angles, accelerations, and rotations and can be used outside the laboratory, making them ideal for real-life gait assessment (both outdoors and at home) [18]. Systems using 3D motion capture, force platforms, and instrumented treadmills have been used for gait assessment in KOA and to identify changes after therapies [19].

### 1.2. Rationale of This Review

Various forms of rehabilitation treatment may offer significant benefits in improving walking impairments for patients with KOA. Prior reviews have addressed conventional clinical assessments and gait analyses in general terms, but these followed a single rehabilitation therapy and did not focus on computerised gait analysis. The use of computerised gait assessment methods avoids potential limitations associated with poor intra- or inter-observer reliability. This review aims to evaluate the effects of different rehabilitation therapies on gait parameters, assessed by computerised methods, with a particular focus on gait speed. Reduced walking velocity is an indicator of functional ability and disease progression, along with increased step-time variability [20]. Slower walking is also associated with increased risks across various clinical and imaging outcomes, highlighting the importance of early identification for preventive intervention [21]. Another purpose is to investigate whether specific therapeutic approaches are associated with improvements in gait speed and to apply them in daily practice.

Regarding improvements in gait speed in patients with knee osteoarthritis (KOA) following various rehabilitation treatments, a systematic review by Fischer M. et al., published in 2019, concluded that whole-body vibration therapy (WBVT) was effective in improving balance and gait speed in patients with KOA, as well as in those recovering from stroke [22]. Additionally, a meta-analysis by Huang et al. on the improvement of kinematic parameters following different physical therapy interventions is relevant for the present analysis and recommended several therapies [23].

### 1.3. Objective

This systematic review aimed to assess the impact of rehabilitation interventions on gait parameters, specifically addressing the following question: “What is the effect of rehabilitation treatment on gait parameters, when assessed by computerised analysis, in patients with knee osteoarthritis?”. Accordingly, a meta-analysis was conducted focusing on gait speed, using data derived from computerised gait assessments.

## 2. Materials and Methods

This systematic review was performed according to the PRISMA (Preferred Reporting Items for Systematic Reviews and Meta-Analyses) guidelines, designed to ensure adherence to standards of transparency and uniformity in data reporting [24]. The review protocol and required data have been recorded in PROSPERO (International Prospective Register of Systematic Reviews) under the following number: CRD420251069722.

### 2.1. Search Strategy

To identify relevant articles on gait improvements in patients with KOA after rehabilitation treatment, assessed using computerised tools, five databases were searched: PubMed, Scopus, Web of Science, Cochrane, and PEDro.

Studies in English published between 2015 and 2025 were selected for this review.

Our study selection was based on the PICO strategy (Population, Intervention, Comparator, and Outcome). The population included patients who were diagnosed with primary knee osteoarthritis (according to the ACR diagnostic criteria) and aged 40 years or older, with balanced cardiovascular and respiratory functions, and who were able to perform walking tests. Children; adolescents; patients who underwent orthopaedic surgery interventions on the knee, hip, ankle, or foot; or patients with neurological diseases (central or peripheral) were excluded. Intervention was represented by the rehabilitation treatment (hydrotherapy, kineto-therapy, manual therapy, massage, physiotherapy, orthosis, or diet). Comparators were the control groups (no treatment, placebo, or other types of treatment). The desired outcome was changes in computer-analysed gait parameters, such as gait speed, walking cadence, KAM, KAAI, GRF, flexion, or extension knee angles during gait stages.

Starting from the study question, the search strategy was designed to identify studies involving patients with knee osteoarthritis who were assessed using computerised gait analysis tools and underwent rehabilitation treatment. The combinations of search terms that were used to identify the studies of interest for this review are presented in Table 1, along with the number of records retrieved for each search sequence.

This search resulted in 2143 articles, which have been archived, organised, and had duplicates eliminated using the ZOTERO reference management software [25].

### 2.2. Study Selection

The selection of studies was conducted by two independent evaluators working separately, following the established inclusion and exclusion criteria. If there were any differences in opinion, a third reviewer clarified the situation. Initially, the selection was based on the article title and abstract; afterwards, articles that appeared to meet the eligibility criteria were read in full.

Inclusion criteria:

- Articles written in English;
- Articles published between 2015 and 2025;
- Randomised controlled trials;
- Studies including patients who were diagnosed with primary knee osteoarthritis and over 40 years of age (according to ACR diagnosis criteria) [26];
- Studies evaluating gait changes in patients with KOA before and after a specific rehabilitation treatment;
- Studies assessing walking using computerised devices for gait parameter analysis.

Exclusion criteria:

- Studies available only as abstracts, conference posters or without full data access;
- Studies conducted on non-human subjects;
- Studies conducted on patients with secondary KOA;
- Studies involving patients who have undergone orthopaedic surgical interventions on the knee, hip, ankle, or foot;
- Studies involving patients with neurological diseases (central or peripheral);
- Studies with extremely small sample sizes (<10 participants per group);
- Studies with a follow-up period shorter than 2 weeks.

### 2.3. Data Extraction

Two independent evaluators extracted specific data from the selected articles, and any disagreement was discussed to reach a consensus. Tables containing information about the study’s author, year of publication, number of participants and characteristics of the populations from the interventional and control groups, type of rehabilitation treatment, computerised device used for gait assessment, and walking speed difference between pre- and post-intervention measurements were created. We extracted information on the gait analysis system used in each study, categorising them into distinct groups: 3D motion capture systems that utilise cameras and markers to provide high-precision kinematic data, often combined with motion capture and ground reaction force measurement; inertial sensors, which use wearable devices to capture temporospatial and kinematic data in real-world settings; and instrumented treadmills equipped with embedded force or pressure sensors. Simultaneously, the previously mentioned scales were used to monitor the evolution of pain intensity, functionality, and quality of life in the selected studies.

Detailed information on the measurement methods used in each included study was gathered and reported below, to summarise the types of equipment and testing conditions applied.

### 2.4. Quality Assessment

The quality of the studies that met our inclusion criteria was assessed using the revised tool to evaluate the risk of bias in randomised trials (RoB 2) for systematic reviews, which checks five standardised domains [27].

### 2.5. Statistical Analysis

First, a qualitative synthesis of the selected studies was performed regarding the population, intervention, control group, and outcome involved.

For the meta-analysis of studies with continuous measurements, MedCalc statistics software version 23.2.6 was utilised. Hedges’ g statistic was used as a measure of the standardised mean difference (SMD) under the fixed effects model, which assumes that the studies share a common true effect and that the summary effect is an estimate of this shared effect size [28]. For heterogeneity, the Q-statistic value (the weighted sum of squares on a standardised scale) and the I-squared value (the percentage of observed total variation across studies that is due to real heterogeneity rather than chance) were examined.

Publication bias was evaluated using the funnel plot, along with Egger’s test and Begg’s rank test. In both tests, a low (two-sided) *p*-value (*p* < 0.05) suggests the presence of publication bias.

## 3. Results

After thoroughly searching the databases using the established combination of terms, a total of 2143 articles were found: 168 from PubMed, National Institutes of Health (NIH); 830 from Scopus, Elsevier; 530 from Web of Science; 595 from Cochrane Library; and 20 from PEDro (Table 1).

The searches in different databases were carried out using specific filters, as shown in Table 2.

**Figure 1:**
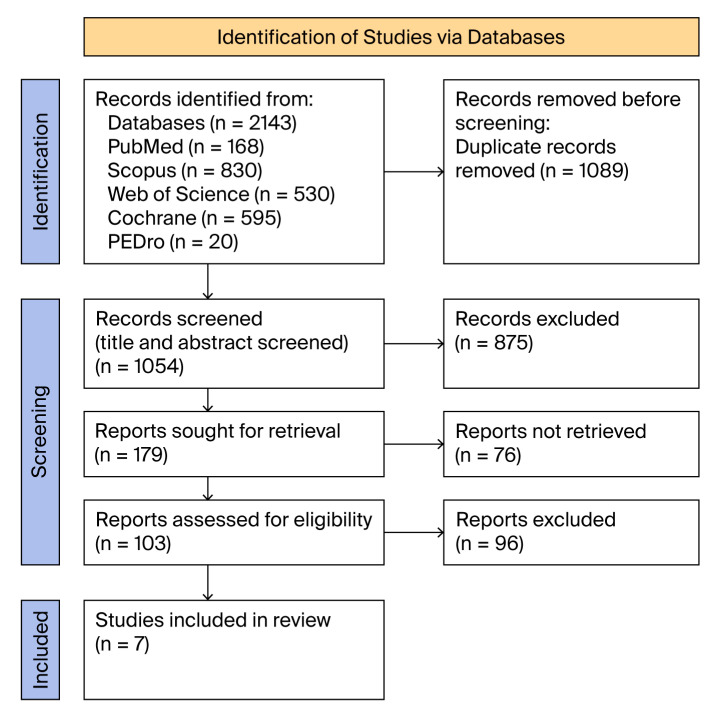
PRISMA flow diagram.

After removing duplicates, 1054 articles remained. After screening titles and abstracts, 875 studies were excluded on the following grounds: not being a KOA study (n = 193), involving surgical intervention (n = 189), ongoing studies (n = 23), not relevant to a rehabilitation intervention (n = 161), not relevant to a gait assessment (n = 147), proceedings (n = 137), or not an original study (n = 25). From the remaining 179 articles, a selection was made after reading each in full, and 76 of the reports were not retrieved. Of the resulting 103 articles, 96 were excluded based on established criteria: prior surgical intervention in the lower limb, inclusion of secondary knee osteoarthritis (KOA), non-randomised controlled trials (RCTs), lack of computerised gait analysis, no gait speed reported, and absence of two walking assessments—before and after the intervention. Finally, seven studies remained, one of which included two interventional groups who were compared with the same control group, resulting in a total of eight randomised controlled trials (RCTs) (Figure 1).

After evaluation, according to our established eligibility criteria, seven studies were included and listed in a table, where the following information was noted: the authors’ names and countries, year of publication, study design, and numbers of participants in the treatment and control groups. Subjects’ ages, body mass indexes, and Kellgren–Laurence [29] radiological stages were also noted (Table 3). According to previous studies, osteoarthritis has been classified into five grades, as follows: No grade (0), Doubtful (I), Minimal (II), Moderate (III), and Severe (IV) [29].

The devices used for computerised gait analysis, type and duration of rehabilitation interventions, and summary of the results were also recorded (Table 4).

### 3.1. Risk of Bias

**Figure 2:**
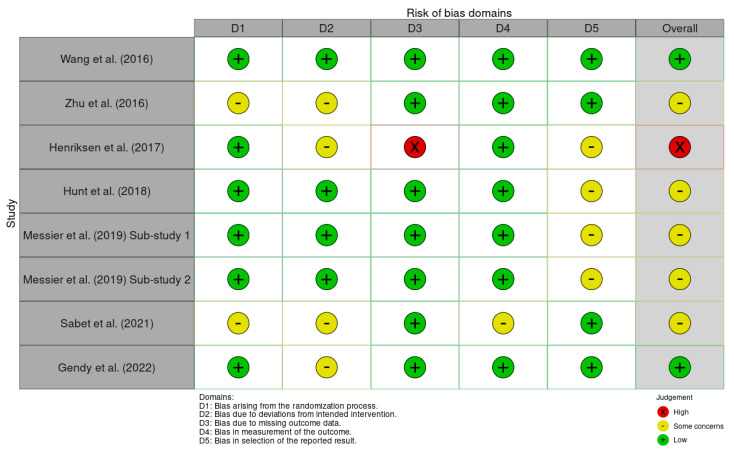
The risk of bias of each study in the 5 domains [30,31,32,33,35,39,40].

Applying the ROB2 tool to all seven studies, we assessed their risk of bias based on computerised gait parameters, expressed numerically. We evaluated the studies according to the five domains and estimated the overall risk by summing their scores (Figure 2). Two assessors separately checked the ROB2-specific questions for each of the five domains. Every discrepancy was noted, discussed, and solved by the third evaluator.

Due to the nature of the interventions (e.g., physical exercise, Tai Ji Quan, treadmill training, diet, WBVT), participant and therapist blinding was not feasible in most studies. However, assessors were blinded in these trials, which minimises the risk of bias in domain 2.

**Figure 3:**
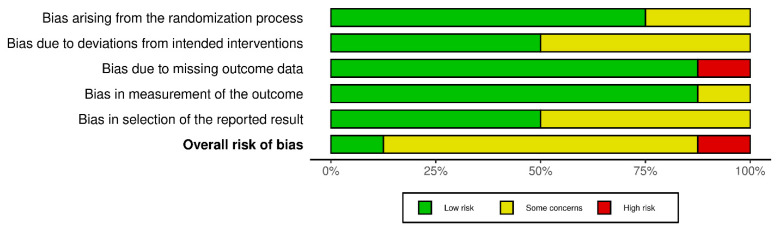
The risk of bias of all the studies.

Most studies were assessed as having some concerns of bias overall, primarily due to issues relating to deviations from intended interventions or outcome reporting, and less regarding the measurement of outcomes and randomisation process. One study was classified as having a low risk of bias, while another was deemed to have a high risk due to significant missing outcome data. Overall, the main source of bias was observed in domains 2 (deviation from intended intervention) and 4 (outcome reporting), which affected our confidence in the evidence. The randomisation and general protocol were well handled overall. The methodological quality was robust, but the absence of protocol registration and selective reporting of data reduced the rigour in several studies (Figure 3).

### 3.2. Description of Included Studies

The study published in 2016 by P. Wang et al. [30] included a total of 39 patients with medial knee osteoarthritis (KOA), divided into two groups: the interventional group (19), who performed Whole-Body Vibration Training (WBVT) combined with Quadricep Strengthening Exercise (QSE), and the control group (20), who only performed QSE. A complex three-dimensional gait analysis was performed while participants walked on level ground at their usual, self-selected comfortable speed. Motion capture was conducted at a sampling rate of 100 Hz using an eight-camera Vicon Nexus system (Denver, CO, USA). A dual-beam photoelectric timing system was used to determine walking velocity, and ground reaction forces were simultaneously recorded using two AMTI force platforms. The WBVT + QSE group showed significant improvements in all spatiotemporal parameters (stance time, swing time, walking speed, cadence, step length, and stride length) after 12 and 16 weeks, with no changes in kinematic and kinetic parameters. The only spatiotemporal feature for which a significantly better enhancement was observed in the interventional group compared with the control group was the walking cadence. Notably, patients who only performed QSE showed improvement in both temporospatial and kinetic and kinematic parameters. Moreover, no significant difference was found between the groups regarding improvement in clinical parameters, such as pain, stiffness, and joint function, even though both showed progress from the baseline [30].

In 2016, Zhu et al. [31] conducted a blinded-assessor RCT involving 46 women who were diagnosed with KOA and aged between 60 and 70 years. The primary aim was to compare two groups (23 participants in the intervention group and 23 participants in the control group) over a 24-week period. The intervention consisted of 60 min Tai Ji Quan sessions three times a week, contrasted with 60 min biweekly educational sessions on gait kinematics in the control group. The outcomes included walking speed, step length, knee flexion angle at initial contact, and peak knee flexion angle during the stance phase. A 16-camera Vicon motion capture system was utilised, with joint centre displacement data being estimated from markers placed on the ankles, knees, and hips. Secondary outcomes were pain, functionality, and balance assessment using WOMAC and the Short Physical Performance Battery (SPPB). Participants who underwent Tai Ji Quan training showed significant improvements in gait velocity, stride length, knee initial contact angle, and peak knee flexion compared with the educational control group, along with better WOMAC and SPPB scores [31].

In their 2017 study, Henriksen et al. [32] evaluated the effects of a functional and individualised neuromuscular exercise therapy programme on walking biomechanics in 60 individuals with KOA (diagnosed according to the ACR criteria) [26]. The study was designed as an assessor-blinded RCT. It included an interventional group of 31 patients, who were assigned to a programme led by a trained physiotherapist three times a week over 12 weeks. The sessions were one hour long and began with a moderate-intensity warm-up on a bicycle ergometer, followed by circuit training exercises designed to improve strength and coordination in the trunk, hips, and knees. Exercises used body weight, resistance bands, or free weights, with individual progression based on a predefined protocol. In contrast, the control group, comprising 29 patients, received no intervention or supervised activity during the 12 weeks. A six-camera, three-dimensional motion capture system (MX-F20, Vicon, Oxford, UK) operating at 100 Hz, synchronised with two force platforms (AMTI OR 6-5-1000, AMTI, USA) that were embedded in the laboratory floor and recording at 1500 Hz, collected kinematic data. A photocell system was used to monitor gait speed, providing real-time visual feedback to the participants via a digital display. The walking speed did not differ significantly between the exercise and control groups. The study found that walking speed, step length, and cadence remained essentially unchanged throughout the intervention [32].

Hunt et al. (2018) [33] published the results of a single-blinded randomised controlled trial (RCT) that included 79 participants (40 in the interventional group and 39 in the control group) with medial knee osteoarthritis (KOA). The intervention involved walking and training with a toe-out angle that was 15 degrees greater than the self-selected angle recorded during the baseline session, following a protocol of verbal instruction and visual feedback in a mirror. For the second group, training sessions included treadmill walking in front of a mirror, following the toe-out protocol, but without the use of foot placement guide tape. Participants’ movements were recorded using a system with twelve high-speed digital cameras (Motion Analysis Corp., Santa Rosa, CA), operating at a sampling rate of 100 Hz, with the aid of twenty-two retro-reflective markers placed on each participant using a modified version of the Helen Hayes marker set [34]. For the kinematic data, the cameras were synchronised with two force platforms (OR6-6, Advanced Mechanical Technologies Inc.). After a 4-month walking programme incorporating toe-out gait modification, significant reductions in knee joint loading (late-stance KAM and KAM impulse) were observed, yielding similar improvements in knee pain and gait speed to those in a walking programme without gait modification [33].

In 2019, Messier [35] et al. published the results of the IDEA study, a single-centre, assessor-blinded, randomised controlled trial conducted over 18 months. Participants were randomly assigned to one of three groups: an exercise-only control (E), an intensive diet-induced weight loss (D) group, and a group subjected to a combination of intensive diet-induced weight loss and exercise (D + E). The study included 454 subjects aged 55 years and older with radiographic evidence of mild or moderate tibiofemoral OA [29], with or without patellofemoral involvement, body mass index (BMI) between 27.0 and 41.0 kg/m^2^, and a sedentary lifestyle, meaning engaging in less than 30 min of structured exercise per week over the past 6 months [36]. Three-dimensional gait analysis was conducted using a 37-marker full-body configuration, in conjunction with a six-camera motion capture system (Motion Analysis Corporation, Santa Rosa, CA) operating at 60 Hz, at baseline and 6-month (FU6) and 18-month (FU18) follow-ups. A six-channel force platform (Advanced Mechanical Technologies, Inc., Newton, MA), integrated with the motion capture setup, recorded kinetic data at 480 Hz. Measured variables included temporospatial parameters (e.g., walking speed, stride length, stride rate) and joint kinematics at the hip, knee, and ankle (flexion/extension, abduction/adduction, internal/external rotation, and ankle dorsiflexion/plantarflexion). Ground reaction forces, inertial components, tibiofemoral compressive and anteroposterior shear forces, patellofemoral compressive force, and associated quadricep, hamstring, and gastrocnemius muscle forces were estimated. At 18 months, both weight loss interventions (D and D + E) significantly reduced knee joint loads compared with exercise alone (E). The D + E group also achieved faster walking speeds, typical of those of healthy adults (1.35 m/s). Importantly, these joint load reductions remained significant even after adjusting the gait speed, highlighting the strong independent effect of a 10% weight loss on knee joint stress. There was no difference between groups in knee abduction moment, aligning with previous findings that tibiofemoral compressive forces are more sensitive to changes in BMI than abduction moment [37]. While diet (D) reduced compressive force and diet plus exercise (D + E) reduced compressive impulses, the abduction moment remained unchanged, possibly due to a narrower step width after weight loss. Additionally, peak knee extension moments after heel strike did not differ between groups at the 18-month follow-up. Although all groups showed improvement from baseline, the values remained below normal, suggesting ongoing quadricep avoidance in patients with knee osteoarthritis [36,38].

Sabet et al. published a study in 2021 [39], designed as a double-blinded, randomised controlled trial (RCT), which included 30 women who were diagnosed with unilateral medial knee osteoarthritis (KOA) (according to the ACR criteria) [26], staged between I and IV Kellgren [29]. Three-dimensional gait analysis was performed using a 3D motion analysis system equipped with six infrared cameras in the biomechanics laboratory. Reflective markers (19 mm) were placed on key lower limb points according to the Plug-in-Gait model. Additionally, the WOMAC questionnaire was used to evaluate pain, stiffness, and physical function of the patients. The intervention group underwent 12 standardised sessions of Swedish massage over 4 weeks (three sessions per week, lasting 20–30 min), applied specifically to the quadricep muscle group of the affected leg, targeting the rectus femoris, vastus medialis, vastus lateralis, iliotibial band, and patellar tendon [39], with no intervention in the control group. Both the intervention and control groups continued their usual medication as prescribed by a specialist, with comparable and equivalent effects. Compared with the control group, patients who received massage therapy showed notable improvements in most spatiotemporal aspects of gait, including a faster walking speed, narrower step width, and higher percentage of total support time, achieved by reducing the initial double-leg support time and increasing the single-leg support time [39].

Additionally, the 4-week Swedish massage intervention resulted in significant clinical and statistical improvements in knee joint stiffness, pain levels, and physical function. These findings align with the outcomes reported in previous clinical trials.

The study by Gendy et al. from 2022 [40], designed as a single-blinded RCT, included 60 patients who were equally allocated into two groups and evaluated improvements in pain intensity, range of motion (ROM), spatiotemporal gait parameters, and function in mild-to-moderate KOA after rectus femoris stretching associated with conventional exercises compared with conventional exercises alone. For gait analysis, the Biodex Gait Trainer 2 (Model 950-380, software), a specialised treadmill system designed for the assessment and rehabilitation of gait disorders, was used. The study showed better progress in terms of VAS, WOMAC, step length, gait speed, and ROM for flexion in the intervention group, but no significant difference in ROM for extension. A relation between ROM and impairment in KOA patients was also noted [40].

### 3.3. Analysis of Included Studies

#### Meta-Analysis: Continuous Measures

Table 5 and Table 6 illustrate our statistical interpretation of the parameters identified in the included studies.

Table 6 lists the results of the individual studies: the number of positive cases, total number of cases, and standardised mean difference with 95% confidence interval (CI) for SMD. If the zero value is not within the 95% CI for SMD, the SMD is statistically significant at the 5% level (*p* < α = 0.05).

**Table 6:** Comparison of numerical data from included studies.

Study	N1	N2	Total	SMD	SE	95% CI	t	*p*	Weight (%)	
									Fixed	Random
Wang 2016	19	20	39	0.362	0.316	−0.279 to 1.003			7.48	12.44
Hunt 2018	40	39	79	0.990	0.236	0.520 to 1.461			13.41	12.60
Gendy 2022	30	30	60	2.601	0.348	1.904 to 3.298			6.18	12.36
Sabet 2021	15	15	30	0.866	0.372	0.103 to 1.629			5.40	12.30
Messier 2019	151	151	302	6.291	0.281	5.739 to 6.843			9.52	12.52
	152	151	303	1.995	0.140	1.719 to 2.271			38.03	12.74
Zhu 2016	23	23	46	0.310	0.292	−0.278 to 0.898			8.81	12.49
Henriksen 2017	31	29	60	0.494	0.259	−0.0248 to 1.012			11.17	12.56
Total (fixed effects)	461	458	919	1.807	0.0866	1.637 to 1.977	20.879	<0.001	100.00	100.00
Total (random effects)	461	458	919	1.740	0.647	0.470 to 3.010	2.690	0.007	100.00	100.00

In the studies by Wang [30], Zhu [31], and Henriksen [32] (, no statistically significant differences were reported between the mean values of the change in walking speed of the two compared groups (*p* > α = 0.05). In contrast, all other studies (i.e., those by Gendy [40], Sabet [39], Messier [35], and Hunt [33], reported significant differences between the two compared groups (*p* < α = 0.05).

The estimated mean effect size is 1.807 (95% CI for SMD: 1.637 to 1.977) and statistically significant (t = 20.879; *p* < 0.001). The estimated Hedges’ g value (1.807) indicates a large effect according to Cohen [41].

The Q-statistic (Q = 353.7297; df = 7; *p* < 0.0001) was found to be significant. The I-squared value (inconsistency) was found to be 98.02% (95% CI for I^2^: 97.24 to 98.58). which may be attributed to differences in group characteristics and sizes, methodologies, and therapies analysed across the included studies. As a result, conducting a comparative evaluation of the treatments is particularly complex.

**Figure 4:**
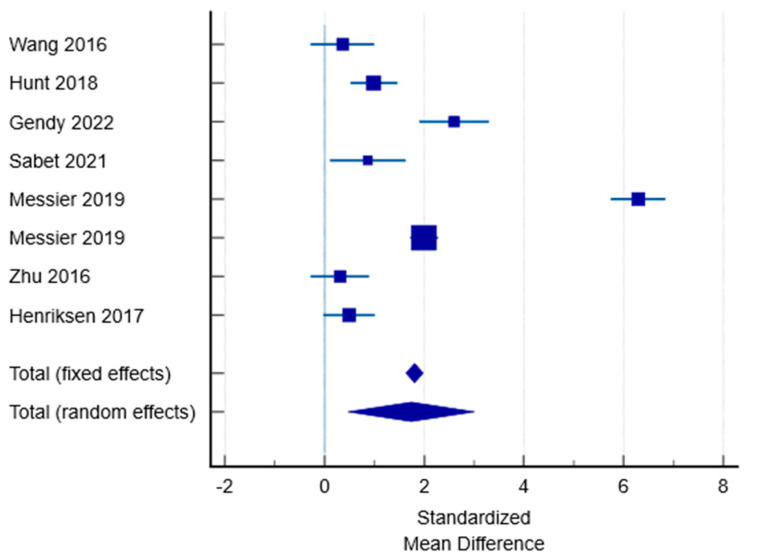
Forest plot of the results of the different studies, with 95% CI, and the overall standardised mean difference with 95% CI [30,31,32,33,35,39,40].

Another method for assessing heterogeneity is the use of a forest plot. As shown in Figure 4, individual studies appear to be distributed heterogeneously.

**Figure 5:**
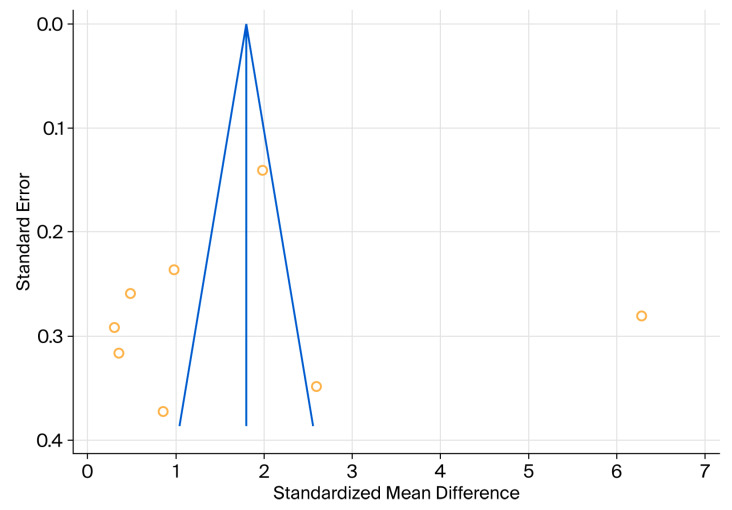
Funnel plot used to assess publication bias.

Egger’s test showed an intercept of −1.3903, with a significance level *p* = 0.8738 and 95% CI: −21.9166 to 19.1360. Considering that the zero value is within the 95% CI and that *p* > α = 0.05, there is no evidence of publication bias (Figure 5). Begg’s rank test assesses whether there is a significant relation between the ranks of the standardised effect size and the ranks of their variances.

Begg’s test uses Kendall’s Tau rank correlation coefficient. Because tau b = 0.07143 and *p* = 0.8046 > α = 0.05, there is no evidence of publication bias based on this test. According to the above results, we conclude that publication bias was not a concern for the examined dataset.

## 4. Discussion

Studies using computerised gait analysis provide more accurate and objective measurements. Previous reviews in the context of patients with KOA have several limitations, including small sample sizes that reduce the robustness of their conclusions; the absence of meta-analyses, which limits quantitative synthesis; and a predominant focus on biomechanical parameters rather than functional outcomes like gait speed, which are more relevant to patient mobility.

Among the various gait parameters, gait speed is considered a valuable and straightforward indicator of survival and quality of life, as it reflects the function of the musculoskeletal system, as well as of the cardiovascular, respiratory, and nervous systems. The walking velocity of a healthy adult ranges between 1.2 and 1.4 m/s, corresponding to 4.3–5 km/h. A speed below 0.8 m/s is associated with an increased risk of falling, hospitalisation, and death, whereas a value under 0.6 m/s indicates functional dependence [42]. A threshold of 1 m/s is necessary to maintain functional independence and safely cross the street [42].

From our meta-analysis, the interventions that displayed statistical significance regarding walking velocity progress compared with control groups are rectus femoris stretching [40], Swedish massage [39], diet-induced weight loss, diet-induced weight loss combined with exercises [35], and guided walking on a treadmill [33]. In the group that underwent diet and exercise, the final mean speed (1.35 m/s) was comparable to that of healthy people.

Not only is the statistical meaning of gait speed improvement important, clinical significance plays an essential role as well. Therefore, out of the seven papers, the increase in walking velocity between baseline and follow-up only surpassed 0.1 m/s in both of Wang’s [30] groups, Gendy’s [40] intervention group one, and Messier’s diet + exercise cohort [35]. This value is considered to be the threshold from which the patient perceives improvement in terms of quality of life [43].

Interventions such as neuromuscular exercises [33] or general rehabilitation programmes without a specific neuromotor component [31] did not lead to clinically significant improvements in walking speed. In Zhu’s study [31] \, the increase from baseline was approximately +0.08 m/s, which is below the MCID threshold, and the lack of other reported clinical or biomechanical parameters reduces the practical relevance of the results. The calculated heterogeneity was high (I-squared value = 98.02%), indicating a high variability between the studies. This substantial heterogeneity severely limits the generalisability of the pooled effect size, as it suggests that individual studies might estimate different true effects from one another. Therefore, the overall estimate should be interpreted with caution, and it may not be appropriate to apply it broadly across different contexts or populations without first investigating the causes of this heterogeneity.

Despite the heterogeneity of the studies assessed in this systematic review, we observed that different types of rehabilitation tools led to improvements in gait speed [33,37,38]. The source of heterogeneity is the variability regarding the sizes of cohorts, the different characteristics of the populations, and the variety of therapies.

### 4.1. Spatiotemporal Parameters

Regarding spatiotemporal parameters, most studies reported statistically significant improvements. The study by Sabet et al. [39] showed notable increases in total support time, along with reductions in step width, initial double-leg support time, and single-leg support time following the application of a Swedish massage protocol. Similarly, Wang et al. reported improvements across all analysed spatiotemporal variables, with positive differences in cadence for the combined WBVT + QSE group [30]. Gendy et al. [40] and Zhu et al. [31] also observed significant increases in step length following rectus femoris stretching and Tai Ji Quan training, respectively.

Conversely, Henriksen et al. [32] reported no significant changes in spatiotemporal parameters or other biomechanical markers, except for isolated differences that were deemed biomechanically insignificant, possibly due to the multiplicity of tests. This absence of effect may reflect the non-specific nature of the intervention or the severity of the disease in the examined cohort.

### 4.2. Kinetic Parameters

Regarding kinetic parameters, Zhu et al. [31] observed a significant increase in the maximum flexion angle and initial contact angle of the knee during the stance phase, suggesting improved joint mobility and normalisation of gait pattern. In contrast, Wang et al. did not identify significant changes in the frontal plane (valgus/varus) or in power generation at the knee and ankle, emphasising that not all exercise interventions influence the fine mechanics of the joint.

### 4.3. Kinematic Parameters

Kinematic parameters were most thoroughly analysed in the studies by Hunt et al. [33] and Messier et al. [35]. Hunt et al. [33] identified a significant reduction in KAM and KAM impulse in the group that was instructed to adopt a “toe-out” gait, suggesting a favourable redistribution of joint load. This gait modification strategy appears to have a more direct and measurable biomechanical impact than general interventions. In addition, Messier et al.’s study showed a significant reduction in tibiofemoral compressive forces and the muscle force required for walking in the groups that were subjected to combined diet and exercise interventions, reinforcing the importance of weight reduction in unloading the knee joint.

Some interventions, such as WBVT, exercises, toe-out gait training, and massage, improved the level of pain and functionality, assessed by standardised scales (WOMAC, SPPB, VAS).

Thus, a trend is emerging, wherein interventions with a specific biomechanical component—whether this is gait modifications or weight reduction through weight loss—generate more consistent effects on kinetic and kinematic parameters. In contrast, generalised exercise programmes or passive interventions (e.g., massage) seem to mainly have significant effects on the spatiotemporal components and subjective symptoms, but less so on the load or alignment parameters. Henriksen et al. [32] and Wang et al. [30] did not show a significant between-group improvement in gait speed. This observation can be linked to the fact that neuromuscular training stabilises the joint and relieves the pain rather than improving the walking performance.

We also mention here a study by Sawada et al. that measured gait velocity at a single time point while patients walked barefoot and with lateral wedge insoles, finding no difference in gait speed between cohorts despite a reduced knee adduction moment in the normal foot group. This study was excluded because of the lack of computerised gait analysis and longitudinal assessment [44].

Sedaghatnezhad et al.’s study from 2019 [45] found that adding uphill treadmill walking to physical therapy for individuals with knee osteoarthritis led to greater improvements in walking speed, measured in a non-computerised manner by the 10-Metre Speed Test, compared with physical therapy alone. The combined approach proved effective, particularly in enhancing and maintaining these functional gains.

L. Varzaityte et al. noted an increased median value after treatment and after a month of its application in interventional groups compared with the controls. They concluded that mineral water baths and mud application improved gait velocity in KOA [46].

The following observation regarding the current guidelines for treating KOA may be worth noting: the European League Against Rheumatism (EULAR) does not include spa therapy, while OARSI mentions it only in the context of generalised OA with comorbidities. According to the updated 2023 EULAR recommendations for the non-pharmacological core management of KOA, all individuals with this condition must be offered an exercise programme that is tailored to their specific needs [47,48,49]. This may involve exercises that target strength, aerobic capacity, flexibility, or neuromotor function, with appropriate dosage and progression based on the patient’s physical abilities, preferences, and available resources. Sessions can be individual or group ones, supervised or unsupervised, and conducted on land or in water, based on local availability and patient preference (class 1A indication). Furthermore, the EULAR recommendations highlight the importance of educating patients on maintaining a healthy body weight to alleviate symptoms and slow disease progression in OA [49]. According to the modified 2019 OARSI recommendations, Level 1B and Level 2 non-pharmacologic treatments for knee osteoarthritis include aquatic exercise, gait aids, cognitive behavioural therapy with exercise, and self-management programmes, which are suitable for patients with or without comorbidities such as GI, cardiovascular issues, widespread pain, or depression [47,48]. Mind–body exercises such as Tai Chi and Yoga are recommended as core treatments with a benefit for patient well-being. Some interventions, including aquatic exercise and gait aids, were downgraded from core to conditional recommendations, as they may not fully align with patient preferences [49].

Henriksen et al. [32] and Wang et al. [30] did not show a significant between-group improvement in gait speed. This observation can be linked to the fact that neuromuscular training stabilises the joint and relieves the pain rather than improving walking performance.

Not only is the statistical meaning of gait speed improvement important, the clinical significance plays an essential role as well. Therefore, out of the seven papers, the increase in walking velocity between baseline and follow-up only surpassed 0.1 m/s in both of Wang’s groups, Gendy’s intervention group one, and Messier’s diet + exercise cohort. This value is considered to be the threshold from which the patient perceives improvement in terms of quality of life.

Thus, a trend is emerging, wherein interventions with a specific biomechanical component—whether this is gait modifications or weight reduction through weight loss—generate more consistent effects on kinetic and kinematic parameters. In contrast, generalised exercise programmes or passive interventions (e.g., massage) mainly seem to have significant effects on the spatiotemporal components and subjective symptoms, but less so on the load or alignment parameters.

The calculated heterogeneity was high (I-squared value = 98.02%), indicating a high variability between the studies. This substantial heterogeneity severely limits the generalisability of the pooled effect size, as it suggests that individual studies might estimate different true effects from one another. Therefore, the overall estimate should be interpreted with caution, and it may not be appropriate to apply it broadly across different contexts or populations without first investigating the causes of this heterogeneity.

### 4.4. Future Research Directions

Future research should investigate biomechanical parameters alongside symptomatic and structural outcomes to gain a more comprehensive understanding of the overall impact of walking on knee osteoarthritis [50]. Comparative studies evaluating diverse rehabilitation interventions—such as muscle resistance training, gait-specific exercises, balance training, and neuromuscular education—are warranted to identify the most efficacious strategies for improving gait speed in this patient population. Furthermore, the implementation of standardised computerised gait analysis protocols is essential to ensure objective and precise measurement, enhance cross-study comparability, and elucidate the specific impact of each therapeutic approach on gait function. In addition, studies involving larger cohorts are necessary to facilitate the development of technologically advanced methods aimed at optimising gait parameters, particularly walking speed, which remains an important determinant of quality of life in individuals with knee osteoarthritis.

Future research also could examine the combined impact of exercise therapy and other treatments, such as manual therapy, electrotherapy, or mud therapy, with gait analysis as the main outcome measure.

### 4.5. Strengths of This Study

This study is strengthened by its rigorous design, conducted in accordance with PRISMA guidelines and based on a comprehensive search of five major databases. A particular strength is the focus on studies utilising objective, computerised gait analysis methods, including 3D motion capture, force plates, and instrumented treadmills. These advanced technologies provide precise, quantifiable, and reliable measurements of gait parameters. Another strength of this study is the implementation of a meta-analysis, which demonstrated a significant improvement in gait speed among patients with knee osteoarthritis following rehabilitation interventions. Although some heterogeneity was present, the overall consistent positive effect across studies supports the clinical relevance of the results, while also indicating that interpretations should consider variability among interventions and populations.

### 4.6. Limitations of This Study

Gait speed consistently showed large and clinically relevant deviations from healthy controls and may be considered as a general marker for gait impairment in knee OA [19].

Each study had its limitations; for example, Zhu et al. [31] only included older women, and the timing of the intervention application was not equivalent between groups. In Sabet’s study [39], women were also included, and massage could be considered a passive method. The main limitation of Hunt et al.’s study [33] is the lack of a control group, while the main one in Henriksen et al.’s study [32] is the small sample size.

There is a high variability between studies, intervention types, population characteristics, and measurement protocols, leading to substantial heterogeneity. Another constraint is the uneven assessment of treadmill walking versus ground walking, as well as the small number of studies (n = 7), which limits the reliability of the general conclusions. Finally, publication bias represents another limitation; the studies reported positive results, and the lack of negative or neutral observations could influence the synthesis.

## 5. Conclusions

Despite the limited number of selected RCTs and the aforementioned limitations, most of them reported significant improvements in walking speed from baseline to the post-intervention period [33,35,39,40]. Four studies noted a significant difference between the intervention and control groups. One study showed a reduction in walking speed following the intervention, with a higher decrease in the control group, suggesting that kineto-therapy may prevent a rapid decline in walking ability.

This review shows that combined interventions, particularly those involving exercise and lifestyle changes (such as weight control), can lead to clinically significant improvements in walking speed in patients with knee osteoarthritis. Only some of the analysed interventions reached the minimum threshold for clinically meaningful improvement, highlighting that statistical significance does not always equate to functional relevance. In addition to walking speed, other spatiotemporal parameters, such as cadence and stride length, can provide important complementary information about functional progress, especially in interventions that do not generate clinically significant speed increases. The high variability in measurement methods, intervention design, and lack of complete data (e.g., absolute values and detailed protocols) limit the robustness of our general conclusions.

## Figures and Tables

**Table 1:** The term combinations used for scoping the international databases.

Term Combinations Used	PubMed	Scopus	Web of Science	Cochrane Library	PEDro	Number of Studies
knee osteoarthritis rehabilitation AND computerised gait analysis	0	1	3	2	0	6
knee osteoarthritis rehabilitation AND gait assessment	24	156	220	114	2	516
knee osteoarthritis rehabilitation AND spatiotemporal gait	3	26	0	13	2	44
knee osteoarthritis rehabilitation AND walking assessment	36	203	0	266	4	509
knee osteoarthritis rehabilitation AND gait inertial sensors	0	18	0	1	0	19
knee osteoarthritis physical therapy AND computerised gait analysis	0	4	0	1	0	5
knee osteoarthritis physical therapy AND gait assessment	19	78	75	80	2	254
knee osteoarthritis physical therapy AND spatiotemporal gait parameters	2	16	17	11	3	49
knee osteoarthritis physical therapy AND walking assessment	36	134	98	4	3	275
knee osteoarthritis physical therapy AND gait inertial sensors	0	1	12	0	0	13
knee osteoarthritis exercises therapy AND computerised gait analysis	0	1	0	1	0	2
knee osteoarthritis exercises therapy AND gait assessment	14	57	20	82	1	174
knee osteoarthritis exercises therapy AND spatiotemporal gait parameters	0	7	3	11	2	23
knee osteoarthritis exercises therapy AND walking assessment	24	111	29	4	1	169
knee osteoarthritis exercises therapy AND gait inertial sensors	0	0	0	0	0	0
knee osteoarthritis AND advanced technology in gait analysis	7	3	21	1	0	32
knee osteoarthritis AND technology in gait analysis AND rehabilitation treatment	3	7	13	3	0	26
knee osteoarthritis rehabilitation AND advanced technology in gait analysis	0	7	13	1	0	21
knee osteoarthritis physical therapy AND advanced technology in gait analysis	0	0	6	0	0	6
knee osteoarthritis exercises therapy AND advanced technology in gait analysis	0	0	0	0	0	0
**TOTAL**	168	830	530	595	20	2143

**Table 2:** Search filters for databases.

Databases	PubMed, National Institutes of Health (NIH)	Scopus, Elsevier	Web of Science	Cochrane Library	PEDro
**Search filters**	2015–present All types of articles English Humans	2015–present All types of articles English Humans All source types and titles of journals	2015–present All types of articles English	2015–present All types of articles English	2015–present All types of articles English
**Number of articles**	**168**	**830**	**530**	**595**	**20**

**Table 3:** Characteristics of study groups.

	Study/Country	RCT Type	No P	No S	No C	Age S + SD	Age C + SD	BMI S + SD	BMI C + SD	K-L
**1.**	Wang at al. (2016) China	Assessor-blinded	39	19	20	61.1 (7.1)	61.5 (7.3)	27.8 (3.1)	6.2 (2.7)	II–III
**2.**	Zhu et al. (2016) China	Assessor-blinded	46	23	23	64.61 (3.4)	64.53 (3.43)	25.23 (3.46)	25.05 (3.42)	I–III
**3.**	Henriksen et al. (2017) Danmark	Assessor-blinded	60	31	29	65.9 (8.5)	61.3 (7.1)	28.7 (4.2)	28.1 (4.5)	I–III
**4.**	Hunt et al. (2018)- Canada	Single- blinded	79	40	39	64.6 (7.6)	65.4 (9.6)	27.3 (3.5)	27.4 (3.5)	II–IV
**5.**	Messier et al. (2019)Sub-study 1 United States of America	Assessor-blinded	302	151	151	66.1 (6.2) (D + E)	66.2 (6.1) (E)	33.6 (3.7) (D + E)	33.5 (3.8) (E)	I–III
	Messier et al. (2019)Sub-study 2 United States of America	Assessor-blinded	303	152	151	65.9 (6.3) (D)	66.2 (6.1) (E)	33.7 (3.6) (D)	33.5 (3.8) (E)	I–III
**6.**	Sabet et al. (2021)- Iran	Double- blinded	30	15	15	52.60 (6.72)	52.40 (6.71)	29.9 (3.37)	29.55 (4.08)	II–IV
**7.**	Gendy et al. (2022) Egypt	Single- blinded	60	30	30	53.63 (6.04)	53.13 (5.94)	53.13 (5.94)	32.06 (0.69)	III

BMI = Body Mass Index; C + SD = Control + Standard Deviation; D = Diet group (interventional); D + E= Diet group + Exercise group; E = Exercise group (control); K-L = Kellgren–Lawrence stages; No. = Number; No. C = Number of patients in Control group; No. P = Total number of Patients; No. S = Number of patients in Interventional group; RCT type = Randomised Controlled Trial type; S + SD = Interventional + Standard Deviation.

**Table 4:** Details on included studies.

Study	Intervention Therapy	Control Therapy	Device for Gait Analysis	Outcome	Treatment Duration	Results	Ref.
**Wang et al. (2016), China**	WBVT + STRETCHING	STRETCHING	System Nexus+ force platforms	VAS, WOMAC Temporospatial parameters Kinetic parameters Kinematic parameters	16 weeks	WBVT + STRETCHING/STRETCHING Improvement in temp-spatial parameters No benefit in kinematics/kinetics Cadence only—better interventional/control STRETCHING Improvements in temp-spat/kinetic/kinematic BOTH GROUPS Improvement—pain/stiffness/function—no diff.	35
**Zhu et al. (2016), China**	TAI JI QUAN 60 min 3/week	EDUCATIONAL SESSION 60 min biweekly	Computerised infrared motion analysis system, a 16-camera setup, and markers	WOMAC, SPPB Gait speed/step length Initial contact angle of the knee Maximal knee angle	24 weeks	TAI JI QUAN/CONTROL Significant improvements: gait velocity/stride length/initial contact knee angle/maximal angle of the knee/WOMAC (pain, stiffness, function)/SPPB	32
**Henriksen et al. (2017), Denmark**	FACILITY-BASED NEUROMUSCULAR EXERCISE THERAPY 3x/week	NO INTERVENTION	Six-camera 3D motion analysis system + force platforms	Joint angles Joint moments Mechanical work Gait speed/step length Cadence Ground reaction force	12 weeks	INTERVENTIONAL/CONTROL Significant difference only in second peak knee flexor moment and second peak vertical ground reaction force No statistical difference—other gait parameters (including speed) Gait speed worsened—both groups	10
**Hunt et al. (2018), Canada**	TOE-OUT GUIDED WALKING ON THE TREADMILL	UNGUIDED WALKING ON THE TREADMILL	Motion capture cam Force platforms Treadmills Biomechanical analysis software Mirror + green tape +protractor device	WOMAC pain/function NRS—intervention KAM KFM Foot progression angle Gait speed	5 months	TOE-OUT/UNGUIDED WALKING ON TREADMILL Significant improvements—knee joint loading Similar improvements—knee pain No improvement—gait speed **(both groups)**	38
**Messier et al. (2019), Sub-study 1 USA**	DIET-INDUCED WEIGHT LOSS + STRUCTURED EXERCISE(D + E)	STRUCTURED EXERCISE SESSIONS WITHOUT DIETARY INTERVENTION(E)	Motion capture system: 6-camera motion analysis system Reflective marker set Force platform, soft	Gait speed Knee joint loading Hip and ankle mechanics Muscle forces	18 months	D + E resulted in significant lower joint loads/E Mean speed (1.35 m/s) (like healthy) No influence on KAM	32
**Messier et al. (2019), Sub-study 2 USA**	INTENSIVE DIETARY WEIGHT-LOSS PROGRAMME (D).	STRUCTURED EXERCISE SESSIONS WITHOUT DIETARY INTERVENTION(E)	Motion capture system: 6-camera motion analysis system, reflective marker set, force platform, software	Gait speed Knee joint loading Hip and ankle mechanics Muscle forces	18 months	D—Lower joint loads compared with E Decreased tibiofemoral compressive force No influence on KAM Peak knee extension moment increased in all groups below normal Peak quadricep muscle force and peak knee extension increased across 3 groups	32
**Sabet et al. (2021),** **Iran**	SWEDISH MASSAGE QUADRICEPS, 20–30 min/ses, 3X/wk	KOA REGULAR TREATMENT	3D motion analysis system: 6 infrared cameras, reflective markers, Cortex analysis software	WOMAC pain/stiffness/function Temp-spatial parameters, including gait speed	4 weeks	SWEDISH MASSAGE/CONTROL Relieved pain Improved function/gait speed/total support time%	36
**Gendy et al. (2022), Egypt**	RECTUS FEMORIS STRECH + CONVENTIONAL EXERCISES	CONVENTIONAL EXERCISES	Universal goniometer, bioindex gait trainer, stopwatch	WOMAC, VAS (pain) ROM-flexion/extension Step length Gait speed	4 weeks	INTERVENTIONAL/CONTROL Improvement—step length/speed gait Higher flexion ROM/no diff. extension ROM Lower VAS and WOMAC scores BOTH GROUPS—better pain/ROM/temp-spat	44

3X/wk = three times per week; D = intensive dietary weight-loss programme; E = structured exercise sessions without dietary intervention; KAAI = knee adduction moment impulse; KAM = knee adduction moment; KFM = knee flexion moment; KOA = knee osteoarthritis; min/ses = minute per session; metres per second; No diff. = no difference; NRS = numerical rating scale; Ref = number of references from each article; ROM = range of motion; SPPB = short physical performance battery; temp-spat = temporal-spatial; VAS = visual analogue scale; WBVT = whole body vibration therapy; WOMAC = Western Ontario and McMaster University Osteoarthritis Index.

**Table 5:** Statistical data from the included studies.

Reference	Treated_N1	Treated_Mean	Treated_SD	Control_N2	Control_Mean	Control_SD
Wang 2016	19	0.17	0.13	20	0.12	0.14
Hunt 2018	40	0.05	0.02	39	0.03	0.02
Gendy 2022	30	0.41	0.12	30	0.16	0.06
Sabet 2021	15	0.12	0.21	15	−0.05	0.17
Messier 2019	151	0.15	0.009	151	0.09	0.01
	152	0.11	0.01	151	0.09	0.01
Zhu 2016	23	0.045	0.1	23	0.015	0.09
Henriksen 2017	31	−0.03	0.04	29	−0.05	0.04

Treated N1=number of patients-interventional group; Treated Mean=average of gait improvement-interventional group; Treated SD=standard deviation of average gait improvement-interventional group; Control N2= number of patients-control group; Control Mean= average of gait improvement-control group; Control SD=standard deviation of average gait improvement-control group.
